# The Birmingham District Nursing Society

**Published:** 1889-11-16

**Authors:** 


### The Hospital Workshop.

### No. XV.—THE BIRMINGHAM DISTRICT NURSING SOCIETY.

(BY OUR SPECIAL COMMISSIONER.)

This is an excellent institution, the type of many that are scattered about the country, more especially in the large towns. By its efforts much real and good work is carried on among the poverty-stricken. The object of the society, to quote from the latest report, is "to supply proper attendance at home to the sick poor whose friends cannot attend to them rightly, and who are unable to pay for a private nurse. This attendance is given gratuitously, but thank offerings from the patients or their friends in aid of the funds of the society will be gratefully received, to enable the committee to enlarge the sphere of its operations," from which the reader will gather that the intention is to provide skilled nursing for the poor in their own homes. The midwifery branch is now given up; there is no undertaking to give any relief beyond the actual nursing, while at the same time chronic or infectious cases are refused. The needed help can be obtained by sending an application to the lady superintendent, on the recommendation of doctors, subscribers, ministers of religion, Scripture-readers, and officers of charitable institutions. Patients are entitled to attendance anywhere within the town of Birmingham. It will be seen, therefore, that a wide extent of ground is included, and in order to limit the action of the society to its proper sphere, no cases are attended excepting under the supervision of a medical man.

The actual work from house to house is done by six nurses, each having her own district. The time occupied by the round is generally from about nine in the morning until three in the afternoon. That finishes for the day, except when there are evening visits to make for taking temperatures or other purposes. The nurses are provided gratuitously by the Birmingham and Counties Training Institute, on joining which each applicant agrees to take any kind of service which may be allotted her during three years, and two of these may be spent in district nursing. A good constitution is required to withstand the latter, for it is undoubtedly very hard work.

Let us join a nurse upon her round. She carries a little black handbag containing a few instruments, bandages, and dressings. At nine o'clock she is already on the way to her district, and, soon afterwards, knocks at the door of her first patient. He is a Scotchman, under treatment from the dispensary, suffering from persistent (and as the event proved, fatal) hæmorrhage from the lungs. He has consumption, and an abscess in the left apex has opened up a bloodvessel, which has been bleeding for a fortnight in spite of every effort to check the flow. The temperature and pulse are to be taken, and a hypodermic injection of ergotine to be given under the skin of the arm. Besides, there is a small threatening bedsore that requires attention. Our next visit is up an alley, where we go upstairs to a tiny bedroom, the stuffiness of which is simply beyond description. Seated in an armchair is the patient, a querulous old Irishwoman in a white cap. She is difficult to manage. For instance, she will not use the ointment ordered by the doctor for her legs, which are badly ulcerated, but insists on using some quack stuff or other with an extraordinary name that she gets from the chemist. Then we go to a tiny shop where oil, and wood, and treacle and such things are sold. Upstairs is one of those distressing cases of senile gangrene of the foot, which is being dressed with iodoform and is doing very well. It is wonderful what a change the use of antiseptics has effected in the treatment of this terrible affection. A few streets off a middle-aged woman is dying of heart disease. Her hands and feet are swollen, her face is blue, and she gasps for breath. In spite of every care bedsores have formed, which require constant attention, and are dressed twice daily. The institution has provided a water-bed, which is simply an invaluable boon under such circumstances. After leaving this patient we go on to a young man who has inflammation of the lungs, and large jacket poultices have to be applied. And so up courts and alleys and streets, in houses clean and in houses dirty, the nurse flits from one to the other until she has finished her list and is ready to go home. During the year 1888 the number of people treated was 794, but that included 118 cases of midwifery, a branch which, as already mentioned, is to be henceforth discontinued. The direct relief to human suffering that is afforded by this unpretending mission is well nigh beyond estimate. Many of the patients would be unable to get admission to a hospital because of the nature of their illness. An analysis of report shows that bronchitis is the commonest illness under treatment. Others come in the following order as to frequency: rheumatism, ulcers, abscesses, cancer and pleurisy, pneumonia, typhoid fever, operations, heart disease. The number of actual visits is of course much larger than the list of patients—namely, 12,314 to the 794 cases. This gives the high average of 15·2 visits per patient, or about 2,052 made by each nurse in the twelve months. That is no light task for any woman to undertake, especially when it is remembered that considerable distances have to be covered daily on foot and in all weathers.

The present home is in a large old-fashioned red brick house in Newhall-street. Inside it is roomy, bright, and cheerful, and seems well fitted to the needs of the inmates. Quite lately a committee of ladies has been formed to go round weekly with the nurses and visit the patients. Personal enquiry of this kind is the only way of preventing abuse of charity, and it seems to place a keystone on the work of the society. The latter is of a deeply interesting character, going straight into the homes of the people and carrying just that kind of help that is needed in the hour of sickness and which cannot be bought with money or at a price.

In conclusion, although it is not customary to refer to matters of this sort, yet in the present case your commissioner feels justified in appending the statement contained in the report, to the effect that "the work of the society is limited only by its subscription list, an appeal for the extension of which is made by the committee."

